# Structural elucidation of estrus urinary lipocalin protein (EULP) and evaluating binding affinity with pheromones using molecular docking and fluorescence study

**DOI:** 10.1038/srep35900

**Published:** 2016-10-26

**Authors:** Durairaj Rajesh, Subramanian Muthukumar, Ganesan Saibaba, Durairaj Siva, Mohammad Abdulkader Akbarsha, Balázs Gulyás, Parasuraman Padmanabhan, Govindaraju Archunan

**Affiliations:** 1 Centre for Pheromone Technology, Department of Animal Science, School of Life Sciences, Bharathidasan University, Tiruchirappalli-620 024, India; 2 Research Institute in Semiochemistry and Applied Ethology (IRSEA), Quartier Salignan, 84400, APT, France; 3 Centre for Animal Research, Training and Services (CAReTS), Central Inter-Disciplinary Research Facility (CIDRF), Mahatma Gandhi Medical College and Research Institute (MGMC-RI) Campus, Puducherry-607403, India; 4 National Centre for Alternatives to Animal Experiments, Bharathidasan University, Tiruchirappalli-620 024, India; 5 Department of Environmental Biotechnology, Bharathidasan University, Tiruchirappalli-620 024, India; 6 Department of Food Science and Nutrition, College of Food Science and Agriculture, King Saud University, Riyadh, Kingdom of Saudi Arabia; 7 Lee Kong Chian School of Medicine, Nanyang Technological University-636921, Singapore

## Abstract

Transportation of pheromones bound with carrier proteins belonging to lipocalin superfamily is known to prolong chemo-signal communication between individuals belonging to the same species. Members of lipocalin family (MLF) proteins have three structurally conserved motifs for delivery of hydrophobic molecules to the specific recognizer. However, computational analyses are critically required to validate and emphasize the sequence and structural annotation of MLF. This study focused to elucidate the evolution, structural documentation, stability and binding efficiency of estrus urinary lipocalin protein (EULP) with endogenous pheromones adopting *in-silico* and fluorescence study. The results revealed that: (i) EULP perhaps originated from fatty acid binding protein (FABP) revealed in evolutionary analysis; (ii) Dynamic simulation study shows that EULP is highly stable at below 0.45 Å of root mean square deviation (RMSD); (iii) Docking evaluation shows that EULP has higher binding energy with farnesol and 2-iso-butyl-3-methoxypyrazine (IBMP) than 2-naphthol; and (iv) Competitive binding and quenching assay revealed that purified EULP has good binding interaction with farnesol. Both, *In-silico* and experimental studies showed that EULP is an efficient binding partner to pheromones. The present study provides impetus to create a point mutation for increasing longevity of EULP to develop pheromone trap for rodent pest management.

Recognition of social responses and sexual advertisement are greatly mediated by chemosignals, and the chemical information has signaling properties that are communicated among members of the same species through pheromones^1^,^2^. Pheromone communication is a universal attribute of life, and perception of chemical information from the environment is crucial for any living system^3^,^4^. Pheromones are mostly released along with the body exudates such as urine, feces, skin- and vomero-nasal gland secretions, etc. In rodents, the urinary chemical messages are delivered by the abundant proteins excreted in urine, which are collectively known as major urinary proteins (MUPs) or α2u-globulins (α2u)^5^. The proteins bind with low molecular weight pheromones or odorants for their transport, slow and sustained release for purpose of identification of conspecifics, scent marks, and other biological applications^6^,^7^, and the proteins are referred to as pheromone-binding proteins (PBP) or odorant-binding proteins (OBP). The binding protein carries the pheromone molecule to the binding cavity of hydrophobic residues for enhancing longevity of the pheromone^6^. Interestingly, the rodent urine also contains proteins which are not volatile but play role as sex-attractants, e.g., darcin, an 18.89 kDa protein found in male mouse urine, which plays role as a sex attractant and in spatial learning in female mice^8^,^9^.

Chemosignals such as thiazole and brevicomin are present in male mouse urine. It has been reported that transport of these chemical messages occurs when they are bound with MUPs and generate broad-ranging effects in the congeners^10^,^11^,^12^. The pheromone compounds farnesol 1 and 2 identified in the preputial gland of house rat, which secretes volatiles abundantly, are bound to α2u^13^. Female rodents and other mammals excrete some specific volatile compounds in urine during the estrus phase of reproductive cycle. These chemical cues in urine attract and invite the partners from the opposite sex for mating. Here also, PBP acts as a carrier and it belongs to the lipocalin family^14^. Estrus-specific volatiles have been reported in the urine of mouse^15^, rat^16^, buffalo^17^,^18^, horse^19^, and elephant^20^.

The rat MUPs have a lot of structural similarity to the members of lipocalin superfamily^13^,^21^. The members of lipocalin family (MLF) proteins are highly diversified in terms of structure, sequence as well as function and play important roles in transport of pheromones and retinol, olfaction, synthesis of prostaglandins and regulation of immune response. These proteins are classified into two types, according to sharing conserved motifs and diversification, viz., kernel lipocalin (KL) and outlier lipocalin (OL)^22^,^23^. We reported the presence of lipocalin family protein in the urine with female rat during estrus, which is referred to as estrus urinary lipoclain protein (EULP). The EULP protein has a maximum structural identity of 63.36% with fatty acid binding protein (FABP), and the homology-modeled protein was validated^14^.

A computational approach is necessary to find the evolutionary clustering pattern of EULP so as to facilitate group categorization and gene ontology (GO) of MLF protein-protein interactions (PPIs) network and pathway association. The networks will be of help in identifying functional annotation of protein interaction pathways based on similarity with the network of another well-characterized species^24^. It is known that conserved residues of protein might be involved in either structure stabilization or PPIs^25^. The dynamical and conformational properties of proteins and their stability are explored adopting molecular dynamics (MD) simulations. The investigation of structural confirmations can throw light on functional properties of EULP. The stability of the EULP with its binding efficiency of endogenous pheromone compound also needs to be examined through *in-silico* and experimental studies.

Therefore, we performed computational analysis of rodent EULP to generate knowledge about sequence and structural annotation and to find the functional backbone. Hence, the present investigation focused on PPIs network, topology, structural superimposition, dynamic changes in structural conformation, and evaluation of pheromone interactions with EULP adopting *in-silico* study. Further, it is important to evaluate the binding efficiency of farnesol with EULP by fluorescence binding assay. The central objective is that the elucidation of EULP binding efficiency with pheromones would facilitate development of novel methods for rodent pest management.

## Results

### Computational analysis

**Figure 1:**
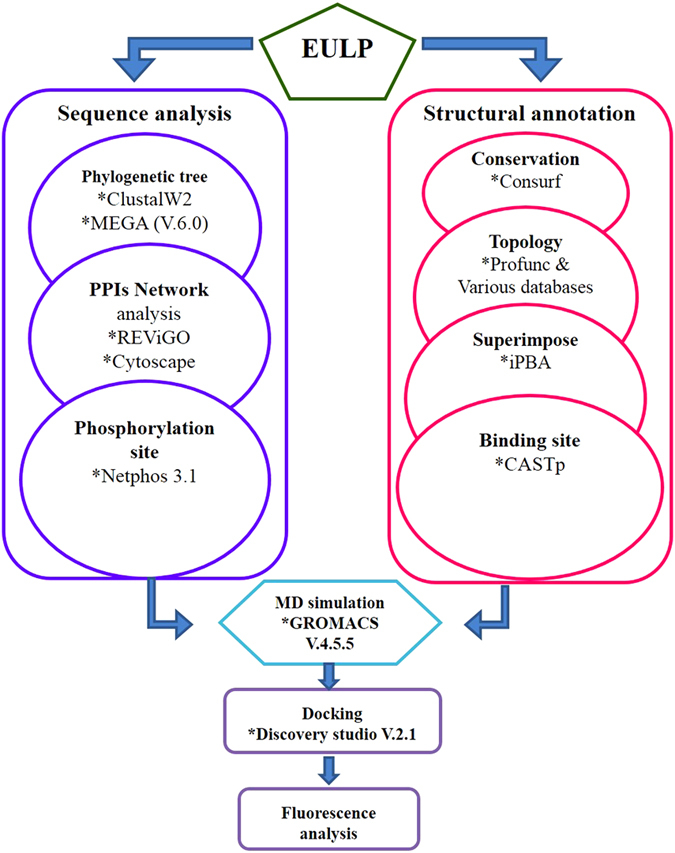
Flowchart depicting the overall workflow. Sequence and structural annotation of EULP adopting computational tools.

The computational analysis consisted of sequence analysis and structural annotation followed by stepwise procedure for MD simulation and docking analysis (Fig. 1).

### Clustering of phylogenetic tree

**Figure 2:**
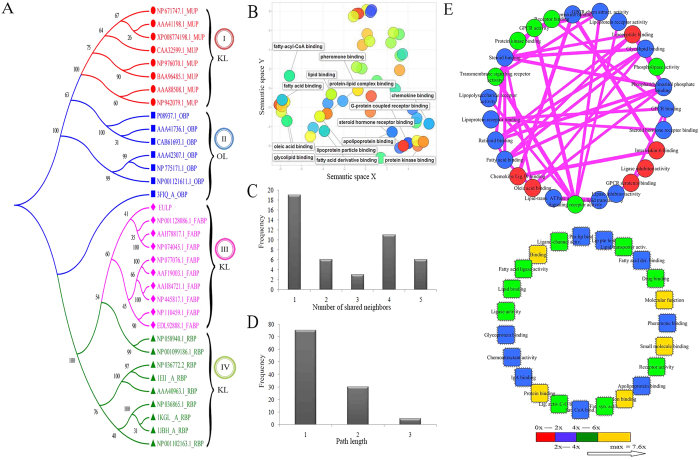
Clustering of phylogenetic tree and protein-protein interactions network. (**A**) Evolutionary relationship analysis of lipocalin family proteins of rat. (**B**) Gene ontology (GO) and network analysis. The scatter plot depicted cluster pattern of binding proteins through REViGO analysis. (**C**) Shared neighbors distribution. (**D**) Shortest path length distribution of network. (**E**) Interacting proteins and Non-interacting proteins network of EULP.

In the present study, the largest sequence resemblances of MLF proteins accumulated in separate clusters and the patterns of four clusters were separated based on the bootstrap (BS) values. The evolutionary analysis revealed that the MUP, FABP and retinol binding protein (RBP) are from KL. Also, the distinct more diversified group of OBP belongs to OL. Additionally, the EULP clade occurs as neighbor cluster (NC) in III^rd^ cluster of rat FABP. Therefore, the preliminary sequence of EULP has the largest percentage of alignment with FABP and confirms that it shares conserved sequence motifs to backbone structural fold (Fig. 2A).

### Gene ontology (GO) enrichment analysis

More than 400 GO entries with p-values were collected and assigned to three major categories viz., biological process (BP), molecular functions (MF) and cellular components (CC). The redundant GO entries were filtered for retrieving a positive dataset. Then GO entries were submitted to reduce visualize gene ontology (REViGO) tool for generating scatter plot of MF (Fig. 2B) and then the proteins were converted to Cytoscape plug-in model for constructing PPI network.

### PPI network of EULP

The PPI network, with 50 nodes and 38 edges, was identified and visualized using Cytoscape (Version.3.2.1). The PPI was characterized and found to have clustering coefficient of 0.3, 1.52 average numbers of neighbors, and the characteristic path length was 1.4. The density of the network was 0.031, heterogeneity 1.3, diameter (∅) 3, and radius (r) 1, without any self loop. The shared neighbors and shortest path length distributions were as shown in Fig. 2CD. The results revealed that the PPI network of EULP could be classified based on log size of proteins as interacting proteins (27) and non-interacting proteins (23). The major interacting proteins were fatty acid-, retinoid-, lipopeptide-, lipoprotein-, steroid-, oleic acid-, GPCR-, glycolipid-, receptor-, and chemokine-binding proteins. The major non-interacting proteins were pheromone-, fatty acid derivative-, glycoprotein-, lipid-, drug-, small molecule-, apolipoprotein-, ion-, and protein-lipid complex binding protein (Fig. 2E).

The integrated proteins datasets were connected by edges to the nodes of proteins, which indicated the molecular interaction between the proteins. The depiction of P-value significance with log size enrichment as different colored parameter scales are red, blue, green, and yellow stripes for 0x-2x, 2x-4x, 4x-6x, and 6x to a maximum of 7.6x, respectively (Supplementary Table S1). The blue and green nodes shows the maximum threshold of the log size for the scale 2x–6x, which correspond to the binding protein.

### Phosphorylation site prediction

The analysis revealed that the EULP sequence has 16 putative phosphorylation sites viz., Ser2, Ser14, Ser32, Ser56, Ser83, Tyr20, Thr8, Thr12, Thr40, Thr54, Thr61, Thr75, Thr84, Thr86, Thr103, and Thr126, respectively. Further, the EULP showed many phosphorylation kinase sites; among them 10 PKC and 2 PKA were included. Further, PKG, CKI, CKII and INSR has each a phosphorylation site (Table 1). The kinas sites are depicted in Supplementary Information Fig. S1A.

### Elucidation of EULP structure

#### Conservation in sequence and structure

The conserved residue analysis of EULP was performed using the ConSurf server. Conservation score was assembled into 1–9 color grade scale. The variable and conserved residue positions present in the EULP were identified. The predicted status of buried (b) and exposed (e) residues showed up in the first row below the sequence. Functionally conserved residues (f) are exposed whereas structurally conserved residues (s) are buried according to neural-network algorithm. The analysis revealed that the predicted functional and structural residues of this protein are highly conserved (Supplementary Table S2). There were 23 residues predicted to be functionally conserved (f) with exposed residues (e) and 4 residues to be structurally conserved (s) with buried residues (b). The sequence and structural backbone conservation score of EULP are shown in Supplementary Information Fig. S1B,C.

#### Topology of EULP

**Figure 3:**
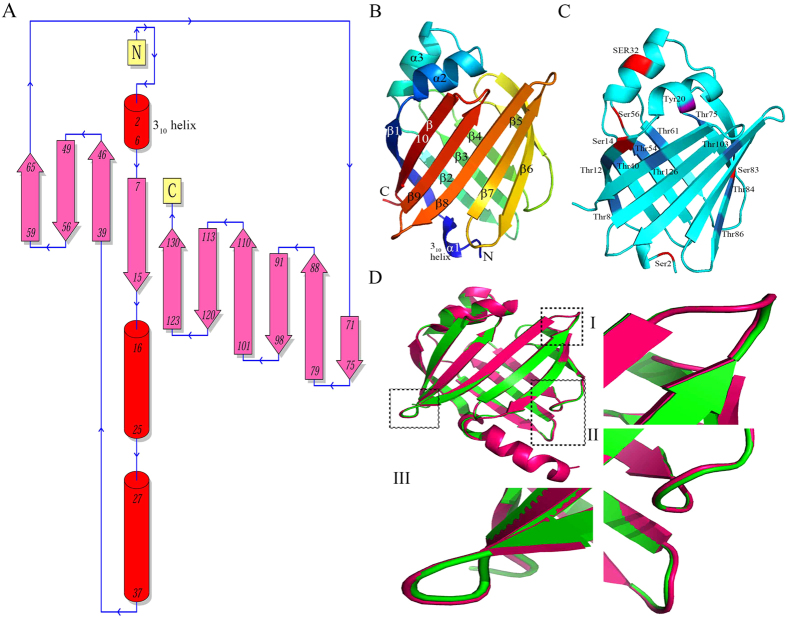
Topology prediction and cartoon representation of structural superimposition. (**A**) Secondary structure based antiparallel topology. (**B**) The β-strands and α-helices are numbered according to their sequence. (**C**) The phosphorylation sites are colored as red, pink and blue for Ser, Tyr, Thr, respectively. (**D**) Structural superimposition of EULP and template showing topologically equivalent positions.

The secondary structure-based topology of EULP was obtained from ProFunc server from European Bioinformatics Institute (EBI) and the backbone structure showed significant anti-parallel topology (Fig. 3A). The secondary structure of EULP scaffold has a 10-stranded β-barrel including approximately 39–130 residues (β-sheet 2–10) connected by loop regions followed by a C-terminal end and the 1^st^ β-sheet followed by 3_10_ α-helices in N-terminal (residues 7–15). The topology of H-bond interaction in EULP is shown in Supplementary Fig. S2A. The EULP showed outside topology, which was identified from TMHMM, HMMTOP and TMPRED servers. It contains two transmembrane segments with critical loop length of 60 residues and the cutoff for putative transmembrane segments score was 0.60, which was identified from TOPPRED method. It was also evident that EULP occurs in non-cytoplasmic cellular location, which was predicted using Phobius (Supplementary Fig. S2B–E). The typical appearance of β-barrel shape and the phosphorylation sites are represented as a cartoon of EULP structure (Fig. 3BC).

#### Superimposition alignment

Structural superimposition was performed for prediction of pair-wise structural alignment between the EULP and FABP (PDB ID: 4A60_A) using improved protein block alignment (iPBA) webserver. The structural similarity score was measured using root mean square deviation (RMSD) between both Cα atom of modeled protein and the corresponding template. The results revealed that the EULP model attained identity of 92.3% for 131 aligned residues with a low RMSD of 0.17Å on their backbone atoms. The quality of alignment showed green color in grade scale, which evidently confirmed the high similarity of the model aligned with template and the global similarity of the two proteins as calculated by global distance test total score (GDT_TS) was 92.2. The cartoon representation of structurally superimposed EULP with FABP comprises the topologically equivalent regions represented as I, II, and III square boxes (Fig. 3D). The analysis revealed that the improved protein blocks (PB’s)-based pair-wise structural alignment could be used to measure RMSD between structurally equivalent Cα atoms of the model for local backbone confirmation and conservation of amino acid composition.

#### Binding site residues

Computed atlas of surface topography of proteins (CASTp) server was used for binding pocket prediction of EULP, which identified and measured as many as of 21 surface active sites with area (A^2^) and volume (A^3^). The potential surface active sites comprise polar atoms in functional region with the highest number of hydrophobic residues. The molecular surface (MS) method was used to measure the area and volume of the protein of interest, which showed 21 binding pockets. Interestingly, the binding pockets 18 and 21 have more hydrophobic residues, in which the phosphorylation site residues such as Ser2, Tyr20, Thr54, Ser56, Thr61, and Thr75, are included (Supplementary Table S3).

### MD simulation of EULP

**Figure 4:**
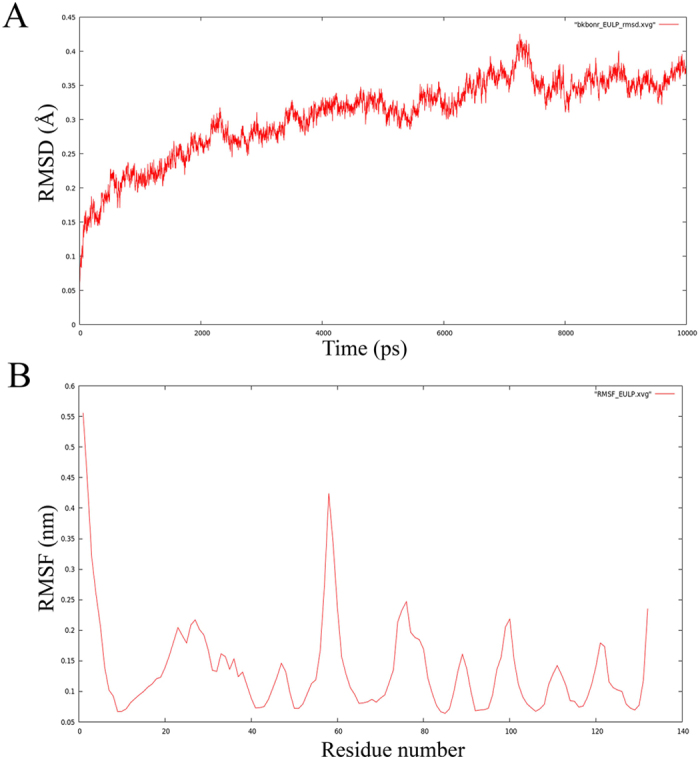
MD simulation of EULP. (**A**) The RMSD profile explains the equilibration nature of the protein throughout the 10 ns MD simulation. (**B**) RMSF plot describes about the EULP has significant fluctuation in the N-terminal residues (Ser2), central coil region (Ser56), and near to C-terminal (Thr86) compared to other residues.

The conformational stability, folding, and dynamic properties of the EULP model were by MD simulation of 10 ns (nanoseconds) duration with different time scales using GROningen MAchine for Chemical Simulations (GROMACS) Version.4.5.5. During MD simulation, the lowest potential energy of the protein ∆E = −5.3611494e + 05 confirmed that the homology-modeled structure was highly stable and the maximum force was E = 9.9024097e + 02 on 967^th^ atom compared to normal force. The EULP structural changes were measured by RMSD with 10000 ps (picoseconds) interval in the whole protein simulation and the changes are denoted in angstrom units (Å). The EULP obtained lowest RMSD below 0.45 Å and the deviation started from below 0.1 Å (Fig. 4A). The root mean square fluctuations (RMSF) were less, and most of the atoms were free from fluctuations. A very few atoms showed RMSF at the N-terminal due to the loop with 3_10_ helix region. We observed the maximum residual fluctuation of above 0.25 nm (RMSF) in the phosphorylation site residues such as Ser2, Thr8, Thr54, Ser56, and Thr61. The other phosphorylation site residues such as Try20, Ser32, Thr75, Thr86 and Thr126 have RMSF values in between 0.2 to 0.25 nm (Fig. 4B). Notably, the N-terminal residue Ser2 has high flexibility in the alignment. Analysis of MD simulation for 10 ns of EULP model was explored which revealed the dynamic behavior and stability of the optimized protein in response to favorable ensemble condition, an aspect suitable for docking study.

### Molecular docking study

**Figure 5:**
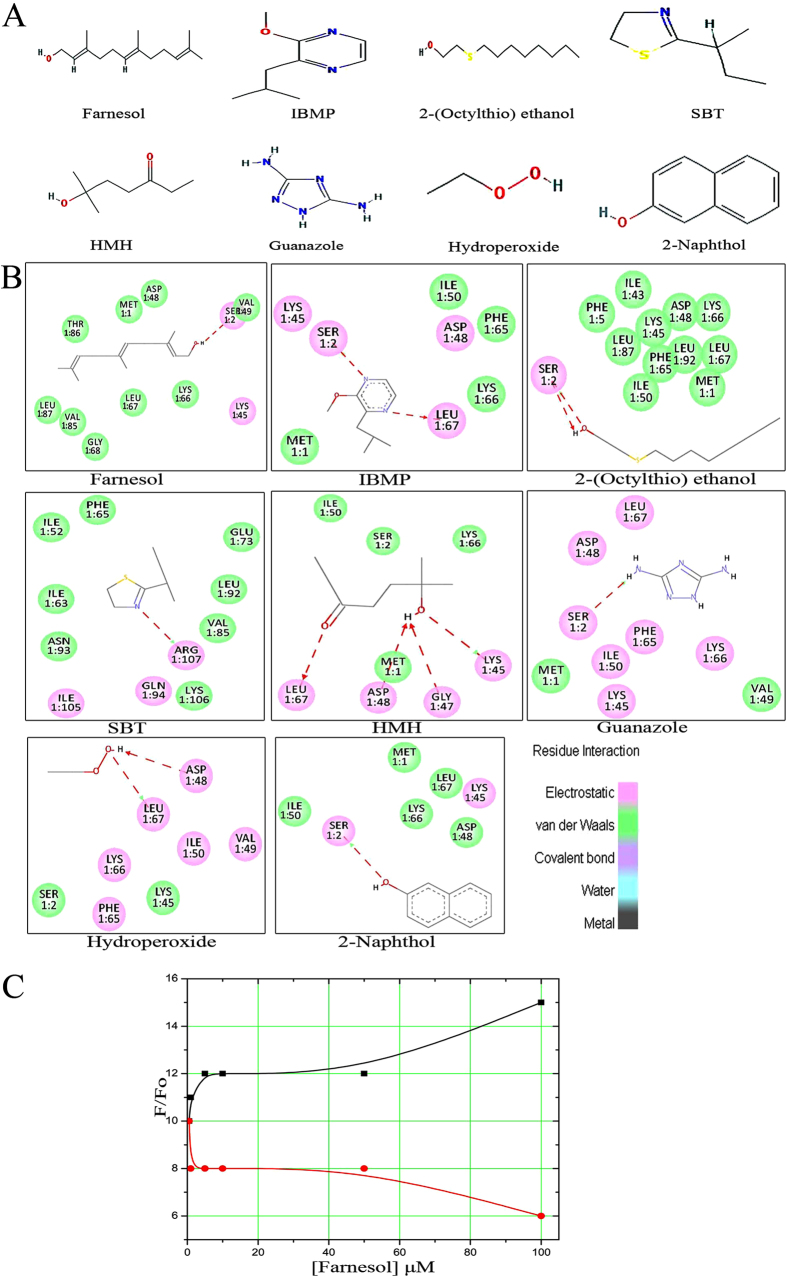
Docking analysis of EULP. (**A**) The selected pheromone/odorant compounds along with the corresponding compound name. (**B**) 2D-diagram of selective compound to the binding sites of EULP; amino acid residues within 4.0 Å to the ligand are displayed. The protein-ligand interactions are depicted with red colored H-bond. (**C**) Competition binding experiments. Fluorescence intensity ratio, F/F_0_ measured at 350 nm and 420 nm. F (black line) represents 2-naphthol presents of increasing concentration of farnesol with EULP and F_0_ (red line) represents the fluorescence of 2-naphthol in the absence of farnesol with EULP.

Protein-ligand interaction analysis is important to identify the best fit suitable ligand from flexible docking of EULP adopting Discovery Studio. The screening procedure is fully based on physico-chemical properties of pheromone compounds. Seven pheromones and one fluorescent compound (2-naphthol) were screened and considered for visual inspection (Fig. 5A). Chemical features, molecular weight (g/mol), and the organism which produces it were used to make a profile for pheromone compounds and fluorescent molecule (Table 2). The evaluation of protein-pheromones-fluorescence interaction was computed mainly in respect of three parameters viz., absolute binding energy, LibDock score and H-bond interactions.

#### Ligand binding poses and energy of EULP

The results suggested that significantly two compounds farnesol (CID_445070) and 2-isobutyl-3-methoxypyrazine (IBMP) (CID_32594) have excellent binding affinity and energy to receptor. Compound CID_445070 showed the highest LibDock score of +92.8, and absolute binding energy of +32.7 kcal/mol, with one H-bond connected with EULP; the oxygen atom of Ser2 formed hydrogen bond with the hydrogen atom of farnesol and the distance was (Ser2-Lig H, 2.9 Å). Compound CID_32594 showed higher LibDock score, binding energy of +60.8 and +32.6 kcal/mol, thus forming two H-bonds with EULP, wherein the first H-bond interaction formed between the HG of Ser2 residue and the N-atom present in the benzene ring of IBMP compound (Ser2 HG-Lig N, 3.1 Å), and a second H-bond interaction was formed between the oxygen atom of IBMP and -NH- atom of Leu67 (Lig O-LEU67 HN, 2.1 Å). Also, the 2-naphthol (CID_8663) has a classical H-bond (Ser2-LigO, 2.8 Å), LibDock score of +59.7 and binding energy of +21.1 kcal/mol with EULP. The residual interactions such as electrostatic and van der Waals forces were represented in pink and green color bubbles (Fig. 5B). The maximum positive value of LibDock score and binding energy signified the highest binding affinity of pheromones with EULP.

Interestingly, the first two compounds, CID_445070 and CID_32594 showed, good binding affinity towards EULP and the LibDock score with binding energy of compounds higher than the other compounds, 2-(octylthio) ethanol (CID_19079); 2-sec-butyl-4,5-dihydrothiazole (SBT) (CID_162148); 6-hydroxy-6-methylheptan-3-one (HMH) (CID_129891), 1,2,4 triazone-3,5-diamine (Guanazole) (CID_15078), hydroperoxide (CID_62472), and 2-naphthol (CID_8663), which were subjected to the docking experiments. The absolute energy, LibDock score, H-bonds and bond length along with steric interacting residues of EULP for the selected compounds is shown in Table 3. Further, the docked sites of the pheromone interaction with EULP were found to be present in the protein surface binding pockets of amino acid residues. The residue Thr86 showed non-bonded hydrophobic interactions and the residue Tyr20 was not involved any interaction towards pheromone since aromatic residues possess less interaction compared to aliphatic residues. Particularly, the Ser2 was the highest interacting residue with H bond/steric bond interactions to pheromone compared to other phosphorylation sites residues as revealed in docking and fluorescent studies. The pheromone interacting residues were almost present in 18^th^ binding pocket of EULP, which are represented in Supplementary Table S3.

### EULP purification

The EULP was purified from urine of female rat using gel-filtration chromatography. The column (length: 28 cm; i.d.: 0.7) was packed with Sephadex G-50, and equilibrated with 10 mM Tris-HCl buffer (pH 7.8). After the equilibration, the concentrated urine was poured onto the top of the column in a single motion and eluted with the same buffer at a flow rate of 1 mL/h. The fractions were analyzed for optical absorbance at 280 nm with a UV visible spectrophotometer and the purity was checked once again by 12% SDS-PAGE. The highly concentrated protein was observed at low molecular mass of 14.5 kDa^14^ and the purified protein (Fraction: 59–63) was used for fluorescence study (Supplementary Information Fig. S3A).

### Competitive binding and quenching

In fluorescence binding study, the emission and excitation states appeared in different concentrations of protein and endogenous compound. Two important fluorescence experiments viz., competitive binding and florescent quenching, were used to evaluate the binding efficiency of protein and ligand (farnesol) with standard fluorescence probe (2-napthol). In competition and quenching experiment, the fluorescence intensity was measured and the results showed that the increasing concentration of farnesol specifies that 2-naphthol is displaced from the protein binding site by farnesol (Fig. 5C). The fluorescence measured as ratio F/F_0_ at 350 nm and 420 nm in competitive binding experiment (where F is the fluorescence intensity in the presence of farnesol and F_0_ is absence of farnesol).

The potassium iodide (KI) quenching analysis revealed that the intensity of endogenous farnesol bound more prominently with EULP, which reflects that increased concentration of linear peak was observed for farnesol with EULP compared to 2-naphthol as ratio F_0_/F measured (Supplementary Information Fig. S3B).

## Discussion

It has been shown earlier that mammals in general, and rodents in particular, release many volatile compounds, which are secreted along with body exudates. These volatiles facilitate recognition of conspecifics towards different physiological and behavioral responses^26^. Female rodents release such volatiles through urine to attract the males for mating. Such volatiles could be used as pheromone trap in the context of rodent pest control. Towards this goal, we also identified proteins that bind and carry these volatiles and found that these odorant binding proteins, belong to the lipocalin family. Thus, we narrowed down, for the first time, to a protein which is present at high concentrations during estrus, and we named it as estrus urinary lipocalin protein (EULP). So, we were interested in characterizing this protein in order to find its evolutionary relationship, function, active site, conserved sites/residues, topology, superimposition, prediction of binding pockets, stability and conformational changes, and the best fit ligand interaction, so as to find the compound which it binds and transports, all making use of bioinformatics tools.

The odorant binding proteins in general and EULP in particular, belong to lipocalin superfamily. MLF proteins are characterized by their molecular recognition properties which reflect on the capability to bind cell surface receptors and small hydrophobic molecules^22^. Towards understanding what our protein of interest, EULP, would most probably bind and transport, we intended to first infer upon the evolutionary relationship with other proteins. So, we embarked on construction of the phylogenetic tree based on mapping of the proteins^27^. The tree was considered in relation to KL and OL and, in case of KL, in relation to MUP, FABP and RBP. Interestingly, the EULP aligned with FABP in the cluster three. Thus, the first inference is that EULP falls under KL in the cluster FABP, which means EULP is a binding protein and what it would bind is a fatty acid. It is of significance because the odorants are by and large fatty acids or their derivatives.

The phylogenetic tree also revealed EULP as distinct from RBP which occurred in the fourth cluster and OL of OBP which aligned with the second cluster. This inference provided lead for further analyzing EULP as an FABP. PPI network provides the valuable framework for perception of the protein functional architecture with interpretation of biological data. Thus, PPI network is useful in deciphering the biological process with which a protein is associated^28^. Therefore, we constructed the PPI network for EULP using Cytoscape plugin and the Data Integration^29^. The PPI network thus constructed for EULP revealed the specific interacting and non-interacting proteins. This network suggested that the edges were inter-connected among fatty acids-, lipoproteins-, retinoic acid-, lipopeptides-, GPCR-, chemokine-, steroid-, and oleic acid- binding proteins, based on the log size. Very specifically, the PPI analysis supported the inference in the evolutionary tree analysis in that EULP is related more with fatty acids and their derivatives, and also GPCR and chemokines.

It is an established fact that PPI analysis throws light on protein-protein interaction which in turn will greatly depend on the phosphorylation sites in the protein of interest. Therefore, we went for phosphorylation site prediction using Netphos 3.1^30^. The analysis led to the prediction that Ser, Thr and Tyr, the putative phosphorylation residues in EULP, along its sequence, and the analysis detected 16 specific sites in EULP for phosphorylation. Thus, the residues Ser, Thr, and Tyr are the potential phosphorylation sites in the protein. Therefore, the EULP has inherent provision for post-translational modification. Further the modification would be useful to predict the various cellular functions regulating protein activity^31^.

The Conseq and Consurf are tools that predict the functionally and structurally conserved residues. Particularly, among the buried (B) and exposed (E) states were 23 functionally conserved and 4 structurally conserved residues were identified. More specifically, Gly7, Trp9 and Phe17 are highly conserved. It is to be noted that Gly and Trp are the most reported -GXW- motif regions. This finding threw indication about the residues which are present in the ligands and/or pheromone binding pockets^32^. The exposition of the conservation pattern is helpful in identification of motif binding sites which would be useful in docking and simulation studies.

Topology prediction is an important step to understand the folds and structural motifs. In this analysis, EULP was revealed to contain 3 helices, 10 strands, 8 beta hairpins, 2 beta bulges, 1 helix-helix interaction, and 14 beta turns. Thus in EULP, beta turns supersede the α-helices. This is an important information for us in the sense the preponderance of beta sheets is clear indication that EULP is not a membrane protein but perhaps an extra-cellular functional protein. The topology prediction adopting ProFunc provided for understanding H-bond interaction within residue-residue contacts^33^,^34^. The information can help in inferring about the function of the protein. The N-and C-terminal location of protein secondary structure was also revealed in this analysis. The superimposition of the EULP modeled protein and template revealed 92.25% identity for 131 aligned residues with a very low RMSD, 0.17 Å, towards the template. Thus, the predicted EULP model and template are greatly aligned structurally, and the template superimposed with model properly. This observation revealed as to how the EULP structure is coordinated to backbone Cα atom of the template crystal structure (4A60_A). Sharing of strong structural folding and conservational local backbone amino acids were also revealed. This can help in further understanding amino acid interaction, contact energy protein of the structure, and geometric quality of the model^35^.

In the next step, we went for binding site analysis adopting CASTp sever, which reveals cavities on protein surface as well as specific amino acid positioning within it to create the physico-chemical properties needed for a protein to perform its function. It also reveals annotated functional information of specific residue on protein structure^36^. It was found that the EULP has several active site residues in 21^st^ binding site identified from area and volume measurements. Further, the results revealed that the 18^th^ and 21^st^ binding pockets are having more hydrophobic amino acid residues compared to other pockets. The hydrophobic residues are capable of hydrogen bond interaction to the corresponding endogenous ligands. Thus, this part of the result substantiates that the modeled EULP is the best one in the light of having many ligand binding pockets. It further substantiated accuracy and structure of the EULP model.

We then subjected the EULP model to analysis of stability and conformational changes at different time scales using MD simulation. The analysis revealed that backbone of the protein has stable structure with lowest RMSD and less RMSF values, which also indicated that the EULP model has the best structural conformation. The MD simulation showed that the energy of the protein was maintained at standard level at the time of simulation and the protein could be used for structure-based drug designing and virtual screening with molecular docking^37^. In the docking study that followed, we screened seven rodent urinary compounds that had ligand interaction with the modeled EULP. The most prominent hydrophobic and hydrogen bond interaction revealed farnesol as well as IBMP as the probable ligands that would bind EULP. Binding assay has shown that farnesol is a good repellent in aphids and the OBP3 and OBP7 are the proteins responsible for mediating the perception of the alarm pheromone^38^. Territorial marking, mating, and individual recognition are the important pheromone effects of farnesol in elephants and various insects^39^. The α and β farnesenes, in male mouse urine, have been reported as pheromones for communication among the congeners^40^. Our study showed that farnesol has the good binding affinity with EULP has revealed in the high LibDock scores compared to other compounds. Thus, the present *in-silico* analysis led systematically through phylogeny construction, GO and PPI analysis, phosphorylation site analysis, conservation analysis, topology prediction, superimposition, binding site analysis, molecular dynamic simulation and molecular docking. It guided us to conclude that EULP is a binding protein which would potentially bind fatty acid or its derivatives. The conserved residue and topology were revealed and the model was proved to be the best fit. Since functionally the protein would bind fatty acid or its derivatives, further analysis led us to infer that farnesol and IBMP would be the most appropriate ligands that would bind with the protein. Farnesol, in a form bound to α2u-globulin, has been identified in the perpetual gland of house rat^13^, and it was also shown to be an alarm pheromone for aphids^38^.

The IBMP is an organic liquid state aromatic odorant having green bell pepper odor. It is insoluble in water and a standard compound used in many competitive binding assays of OBP^41^,^42^. It has been shown that the odorant IBMP binds to specific receptor of cow olfactory mucosa^43^,^44^, and the heterocyclic pyrazine-binding proteins are abundantly present in mammalian nasal mucus which participates actively in transport of such odorant molecules^45^. The present EULP model substantiated the most prominent hydrophobic and hydrogen bond interaction with IBMP as well as in farnesol. Compared to other odoriferous compounds, farnesol has good binding affinity with EULP and has high LibDock score. The residue, Ser2 has maximum flexibility and residual interactions towards pheromone compare to other phosphorylation site residues. Thus, the results demonstrated that Ser2 may be suitable site for post translational modification in EULP and it may act as a crucial functional site for pheromone shuttle.

In order to predict the pheromone interactions of intact EULP, competition and quenching analysis were performed using 2-naphthol with KI as quencher. The fluorescence quenching intensity ratio F_0_/F was measured for 2-naphthol in the presence of EULP at increasing concentrations of farnesol on excitation peak. The binding efficiency obtained by the dissociation constant (K_d_) of EULP with 2-naphthol was measured as K_d_ = 0.42 ± 0.06 *μM* and it is understood that farnesol might possess the same K_d_ as that of 2-naphthol. Therefore, the residue, Ser2 reveals similar interaction and optimal binding energy with farnesol and 2-naphthol. This shows that Ser is a dynamic and capable residue for EULP structure modification and for better interaction with pheromones. This preliminary report demonstrated that farnesol has more hydrophobic affinity with binding pockets of EULP. The present results will facilitate our effort to develop a pheromone trap for use in rodent pest management using farnesol and/or EULP, with the expectation that if farnesol in the trap is bound to EULP there will be slower release of the volatile such that the trap will provide for sustained release of the attractant. Based on the predictions, we propose that chemosignal communication in the rat might be facilitated by EULP, and this can act as a carrier protein so as to decode chemical signals of pheromones and odorants to attract the congeners. The results obtained from this study would facilitate generation of a point mutation in EULP adopting protein engineering technologies so as to increase its longevity and thus to develop a novel pheromone trap for rodent pest management (RPM) program.

## Methods

### Evolutionary relationship analysis

#### Collection of lipocalin sequences of rat

The MLF protein sequences of rat such as MUP, FABP, OBP, and RBP were retrieved from non-redundant protein (nr) database of National Centre for Biological Information (NCBI) (http://www.ncbi.nlm.nih.gov) and protein data bank (PDB) (http://www.rcsb.org). The retrieval of similar proteins was based on iterated profile search with alignment scoring matrix, which was made by position-specific iterated basic local alignment searching tool (PSI-BLAST)^46^. First, around 75 sequences were collected, which were submitted for removing redundant sequences at 99% adopting cluster database at high identity with tolerance by (CD-HIT) server (http://www.bioinformatics.org/cd-hit)^47^.

#### Phylogenetic tree construction

The resulting 34 non-redundant sequences were submitted for analysis of multiple sequence alignment (MSA) executed by GONNET weighted matrix with gap extension in ClustalW2 program (http://www.ebi.ac.uk/Tools/msa/clustalw2)^48^. Further, the sequences were visualized and the unaligned indels from the generated alignment were resized manually adopting MEGA V.6.0 for ascertaining the quality of the alignment^49^. The phylogenetic tree was constructed by computing genetic distance from multiple sequence alignments using distance-matrix statistical method of neighbor joining (NJ) approach and Poisson model for substitution with the 1000 BS replicates. Also, the bootstrap values of MLF proteins were observed by traditional curved phylogeny for better understanding of distribution of clusters.

### GO enrichment and PPI network analysis

Identification of GO ID, enrichment, and elucidation of p-values of EULP sequence were carried out through GO consortium (http://geneontology.org/page/go-enrichment-analysis) connected to PANTHER classification system. The predicted GO term was used to assign proteins to distinct clusters and generate a scatter plot using REViGO analysis that is specific to molecular function (http://revigo.irb.hr)^50^. The tree map of PPI was predicted and retrieved as Cytoscape plugin for generating integrated network. The integrated network was visualized through Cytoscape V.3.2.1, which is an open source software platform supported with Java environment to develop complex biological networks^51^. Also, Cytoscape adopts nested network style with log size-based group attributes for establishing circular layout of PPI network.

### Phosphorylation site

Phosphorylation sites and kinase-specific phosphorylation sites such as Ser (S), Thr (T) and Tyr (Y) of EULP sequence were predicted using the online web servers NetPhos 3.1 (http://www.cbs.dtu.dk/services/NetPhos-3.1/)^30^. The program works based on artificial neural network algorithms.

### Structural documentation

#### Conserved residue analysis

The EULP model was analyzed for the identification of functional residues and mapping conserved regions in both exposed and buried residues of structure using ConSeq and ConSurf tool (http://consurf.tau.ac.il/)^52^. This is an online web server, which calculates conservation score of the position of individual amino acids among their close sequence homologues using Bayesian algorithm, and the default JTT matrix was used as evolutionary substitution model. The Consurf algorithm was used to evaluate confidence intervals of calculated conservation score visualization based on 1–9 grade scale depicting variation in color. The most variable residue positions were present in grade 1 (turquoise) and the most conserved residue positions were present in grade 9 (dark red). Accordingly, the protein structure was also colored and it was visualized by JSmol (default mode for First glance).

#### Topology prediction

The prediction of EULP structural topology was carried out using various web-based online servers such as ProFunc (http://www.ebi.ac.uk/thornton-srv/databases/profunc)^53^ from EBI, TMHMM, HMMTOP, TMPRED, TOPPRED, and Phobius for getting a prominent fold architecture of the target protein. The multiple web servers were used to compute the inside and outside topologies of the N-terminal residue and the prediction of hydrophobicity plot for analysis of transmembrane (TM) region in the protein.

#### Structural superimposition of EULP

Homology-modeled structure of EULP and its template structure (PDB: 4A60_A) were superimposed using iPBA (http://www.dsimb.inserm.fr/dsimb_tools/ipba/index.php). This algorithm was used for pair-wise comparison of multiple structures and it employs anchor-based dynamic programming for identifying structurally conserved regions as local alignments. The degree of structural similarity between backbone Cα atom pairs of the model and the respective templates was calculated by RMSD. The iPBA was extensively outperformed using a nontrivial benchmark dataset and compared to other well established structural alignment methods^36^.

#### Binding site residue

The active site of the modeled EULP was analyzed using CASTp (http://sts.bioe.uic.edu/castp/calculation.php) online web server^36^. It is used to identify and measure concave surface features (i.e., area and volume) with functional regions of proteins. The active site residues were characterized by two parameters, namely the solvent accessible surface (SA, Richards’ surface) and the molecular surface (MS, Connolly’s surface). The predicted active site residues were used to confirm the binding pocket for further protein-ligand interaction studies.

### Protein model submission

The 3D coordinates of homology-modeled protein was structurally documented and deposited in Protein Model Database (PMDB) (https://bioinformatics.cineca.it/PMDB/). The database provides information about manually built homology-modeled proteins in a manner simple to deposit or retrieve the model in PMDB. The EULP model was deposited successfully and accepted in PMDB. After submission, a unique identification number **(PMDB ID: PM0079851)** was provided by PMDB for the EULP model.

### Simulation of EULP structure

The MD simulation was carried out for the modeled EULP using GROMACS V.4.5.5 (http://www.gromacs.org/)^37^ software package to analyze the stability of the protein. The MD simulation of EULP was performed using GROMOS96 53a6 force field with twin-range cut off (0.9/1.44 nm) for default treatment of electrostatics. The protein was solvated with flexible simple point charge (SPC) water model, which added 11050 SOL molecules to system for aqueous surroundings. The preliminary structure was immersed as the center part in a cubic box maintaining 1.0 nm distance allowed between the edge of the box and the protein surface. The system has 2.00 as non-zero total charge, and it was neutralized by replacing 2 CL– counter ions instead of 2 solute molecules in topology file. Steepest descents algorithm is a first-derivative method and it was employed for energy minimization, which converged to fmax <1000 in 268 steps. The molecular bond lengths and geometry of the water were constrained with linear constraints solver (LINCS)^54^ and SETTLE^55^ algorithm, respectively. In order to regulate the temperature (310 K) and pressure of the system, the V-rescale weak coupling and Parrinello-Rahman method, respectively, were used. The particles mesh ewald (PME) method was used to analyze long range electrostatic interaction, and the estimated of PME mesh part was 0.31. Cutoff distances for the coulomb and van der Waals interaction were 0.33, and 0.3 nm, respectively. Fourier grid dimensions of 60 × 60 × 60 with 0.118 spacing each was calculated for X, Y, and Z, respectively. The ensembles of both NVT (constant number of molecules, volume and temperature) and NPT (constant number of molecules, pressure and temperature) were used for 100 ps. In continuation, this pre-equilibrated system was employed to 10 ns (i.e., 10000 ps) production MD simulation at intervals of 2 femtoseconds (fs). Every 2.5 ps simulation was ensured to analyze the energy stabilization plots, RMSD with RMSF in EULP and the least potential energy was preferred for further docking studies. Visual Molecular Dynamics (VMD) (http://www.ks.uiuc.edu/Research/vmd/) graphics software was used for visualization of the EULP simulation outputs.

### Molecular docking

#### Collection of compounds

The structural coordinates of about 32 endogenous pheromone compounds (ligands) were retrieved from PubChem (https://pubchem.ncbi.nlm.nih.gov/) and, then from these, 11 compounds were filtered and eliminated to retrieve the naturally secreted rodent pheromones and odorants in relation to the organisms which produce them. The selection was narrowed down to 7 compounds via Lipinski rule of five (RO5) web server (http://www.scfbio-iitd.res.in/software/drugdesign/lipinski.jsp) and docking experiments. The selected ligands are Guanazole^16^; IBMP^42^; 2-(octylthio) ethanol^16^; SBT^56^; HMH^57^; farnesol^40^; and hydroperoxide^16^; the ligands were included for retrieval of information about compound category, state, odor, and solubility as shown in Supplementary Table S4. Also, the fluorescent compound 2-naphthol was employed to find a binding affinity and residual interaction towards EULP using docking. Tanimoto similarity analysis expounds that the 3D structures of farnesol and 2-naphthol are not similar (Supplementary Information Fig. S3C).

#### Physico-chemical property analysis and Optimization of ligand

Physico-chemical properties of all ligands were identified from various databases such as PubChem, ChemSpider, and DrugBank (Supplementary Table S5). In addition, the physico-chemical properties of pheromone compounds which included H-bond donor-acceptor, topological polar surface area (A^2^), density, boiling, melting point, octanol/water partition coefficient and Lipinski RO5 were tabulated and presented in Supplementary Table 5. The energy minimization of EULP and all ligands were prepared in Swiss-PDBViewer adopting GROMOS96 Force Field algorithm.

#### Protein-ligand complex

Interaction of novel endogenous ligands with the EULP model was analyzed by docking module in Discovery Studio. Accelrys Software Inc (V.2.1) (Accelrys, San Diego, USA) was used for molecular docking studies (http://accelrys.com/products/discovery-studio/).

### Experimental animals

The female house rats (*Rattus rattus*) were trapped at residential areas close to Bharathidasan University, with proper permission from the house owners. The reproductively active female rats (150–200 g) were selected and acclimatized to laboratory conditions for a month. The rats were housed in polypropylene cages (40 × 25 × 15) with 2 cm rice husk lining at the bottom as hygienic bedding material, which was changed once every three days. The regular estrous cycling animals were selected for further experimental study. All animals were maintained in 12:12 L & D cycle throughout the experimental period. All the experimental procedures were followed according to the guidelines of Institutional Animal Ethics Committee (IAEC) and also the experimental protocols were approved by Bharathidasan University, Tiruchirappalli.

### Urine collection and purification of EULP

The urine was collected from female rats (n = 20), maintained at metabolic cage for three consecutive estrous cycle and then the urine samples were prepared according to Muthukumar *et al*.^14^. Then the EULP was purified using size exclusion chromatography method according to Cavaggioni *et al*.^58^. The urinary protein fractions were analyzed using UV-visible spectrophotometer (Systronics, India) at 595 nm and the purity was checked by SDS-PAGE^59^.

### Fluorescence study

In this study, 2-naphthol was used without further purification and the concentration of EULP protein (24 μM), and 2-naphthol (1 mM stock) were prepared in 100% ice-cold ethanol, which was stored in the dark at 4 °C. The 2-naphthol was further diluted for each experiment to a final concentration of 25 μM and the 10 mM phosphate buffer used at pH 7.2 at room temperature in all experiments. All pH values were measured by a digital pH meter with a combined glass-calomel electrode (Systronics, India). Fluorescence intensity and the measurements were carried out using Synergy *HTX* multi-mode microplate reader (BioTek Instruments, Inc., Winooski, VT, U.S.A) and FP-6500 spectrofluorometer. Fluorescence determinations were made using a tungsten halogen lamp with a PMT detector for maximum sensitivity and the data collection, analysis of results, and exporting were done by Gen5 Data Analysis Software.

#### Competitive binding and quenching

We were interested in analyzing the binding efficiency of EULP with endogenous pheromone, farnesol (IUPAC: (2E, 6E)-3, 7, 11-trimethyldodeca-2, 6, 10-trien-1-ol) using competition experiments. The farnesol stock solution was 0.7 mM in ice cold ethanol and it was used to displace 2-naphthol from the binding site of EULP. The fresh 2 M solution of KI was used as quencher in fluorescence analysis^3^.

## Additional Information

**How to cite this article**: Rajesh, D. *et al*. Structural elucidation of estrus urinary lipocalin protein (EULP) and evaluating binding affinity with pheromones using molecular docking and fluorescence study. *Sci. Rep.* **6**, 35900; doi: 10.1038/srep35900 (2016).

**Publisher’s note:** Springer Nature remains neutral with regard to jurisdictional claims in published maps and institutional affiliations.

## Supplementary Material

Supplementary Information

## Figures and Tables

**Table 1:** Prediction of putative phosphorylation site in EULP sequence.

S. No	x	#	Context	Score	Kinase	Answer
1	S	2	MSNKFL	0.52	PKC	YES
2	T	8	KFLGTWKLT	0.92	PKC	YES
3	T	12	TWKLTSSEN	0.59	PKG	YES
4	S	14	KLTSSENFD	0.58	CKII	YES
5	Y	20	NFDEYMKAL	0.59	INSR	YES
6	S	32	LGTRSLGNL	0.77	PKA	YES
7	T	40	LAGPTVIIS	0.73	PKC	YES
8	T	54	ITIRTESGF	0.51	PKC	YES
9	S	56	IRTESGFKN	0.81	PKC	YES
10	T	61	GFKNTEISF	0.65	PKC	YES
11	T	75	FEETTADNR	0.51	CKI	YES
12	S	83	RKTKSTVTL	0.56	PKA	YES
13	T	84	KTKSTVTLA	0.82	PKC	YES
14	T	86	KSTVTLAGG	0.64	PKC	YES
15	T	103	NGNETTIKR	0.72	PKC	YES
16	T	126	SVVCTRIYE	0.63	PKC	YES

NetPhos score is the output from the ensemble of neural networks trained data. Description for #-the position of the residue, x-the residue in one-letter code, Answer- the string “YES” for positive prediction.

**Table 2:** Collection of putative pheromone ligands and fluorescent 2-Naphthol from PubChem database and the pheromones were selected based on the source of organisms.

S. No	Pubchem ID	Compound Name	Chemical formula	Molecular weight (g/mol)	Organisms	Source
1	CID_15078	guanazole	C_2_H_5_N_5_	99.09	Male rat	Urine
2	CID_19079	2-(octylthio) ethanol	C_10_H_22_OS	190.34	Male rat	Urine
3	CID_32594	2-isobutyl-3-methoxypyrazine (IBMP)	C_9_H_14_N_2_O	166.22	Male rat	Urine
4	CID_162148	2-sec-butyl-4,5-dihydrothiazole (SBT)	C_7_H_13_NS	143.25	Male mouse	Urine
5	CID_129891	6-hydroxy-6-methylheptan-3-one (HMH)	C_8_H_16_O_2_	144.21	Male mouse	Urine
6	CID_445070	farnesol	C_15_H_26_O	222.36	Male rat & mouse	Preputial gland & Urine
7	CID_62472	hydroperoxide	C_2_H_6_O_2_	62.06	Female Rat	Urine
8	CID_8663	2-naphthol	C_10_H_8_O	144.16	—	—

**Table 3:** Ligand’s interaction toward EULP.

S. No	Compound name	Binding energy kcal/mol	LibDock Score	H-Bonds	Bumps or Poses	Residue involved in Hydrogen bond	Bond length distance (Å)	Steric interaction	
								Bonded	Non-bonded
1	farnesol	32.7	92.7	1	10	Ser2	2.9	Met1, Ser2, Asp48, Leu67, Leu87	Lys45, Gly47, Val49, Ile50, Lys66, Val85, Thr86, Ala88
2	IBMP	32.6	60.8	2	13	Ser2	3.1	Asp48, Lys45, Ile50, Leu67	Met1, Gly47, Val49, Phy65, Lys66
						Lue67	2.1		
3	2-(octylthio) ethanol	18.1	59.9	2	73	Ser2	2.5	Ser2, Lys45, Asp48, Ile50, Leu67, Leu92	Met1, Phe5, Ile43, Val49, Phe65, Lys66, Leu87
						Ser2	2.3		
4	SBT	17.1	56.9	2	98	Arg107	2.2	Ile43, Leu92, Gln94, Ile105, Arg107	Ile52, Phe65, Glu73, Val85, Asn93, Lys106
5	HMH	8.9	51.5	3	97	Lys45	2.8	Lys45, Asp48, Leu67	Met1, Ser2, Lys45, Gly47, Val49, Ile50, Phy65, Lys66
						Gly47	2.6		
						Asp48	2.2		
						Lue67	2.3		
6	guanazole	13.1	45.1	1	9	Ser2	3.5	Lys45, Asp48, Ile50, Leu67	Met1, Gly47, Val49, Phe65
7	hydroperoxide	1.9	29.9	2	29	Leu67	2.8	Asp48, Ile50, Lys66	Ser2, Lys45, Val49, Phy65
						Asp48	2.8		
8	2-naphthol	21.1	59.7	1	21	Ser2	2.8	Lys45	Met1, Asp48, Ile50, Lys66, Leu67

Notably, Protein-Ligand interactions observed and identified best interacting ligand based on binding energy and LibDock score of ligands towards EULP.
