# Supplementary material for: Structural elucidation of estrus urinary lipocalin protein (EULP) and evaluating binding affinity with pheromones using molecular docking and fluorescence study
Source: Sci Rep. 2016 Oct 26;6:35900. doi: 10.1038/srep35900 (PMC5080580; doi:10.1038/srep35900)
Supplement: Supplementary Information [file srep35900-s1.pdf]

Structural elucidation of estrus urinary lipocalin protein (EULP) and evaluating binding affinity with pheromones using molecular docking and fluorescence study

Names and affiliations of authors:

Durairaj Rajesh[1,2], Subramanian Muthukumar[1,3], Ganesan Saibaba[1,5], Durairaj Siva[4], Mohammad Abdulkader Akbarsha[5,6], Balazs Gulyas[7], Parasuraman Padmanabhan[7]*, Govindaraju Archunan[1,5]*

[1] Centre for Pheromone Technology,
Department of Animal Science,
School of Life Sciences,
Bharathidasan University,
Tiruchirappalli- 620 024, India.

[2] Research Institute in Semiochemistry and Applied Ethology (IRSEA),
Quartier Salignan, 84400, APT, France.

[3] Centre for Animal Research, Training and Services (CAReTS),
Central Inter-Disciplinary Research Facility (CIDRF),
Mahatma Gandhi Medical College and Research Institute (MGMC-RI) Campus,
Puducherry- 607403, India

[4] Department of Environmental Biotechnology,
Bharathidasan University,
Tiruchirappalli – 620 024, India.

[5] National Centre for Alternatives to Animal Experiments,
Bharathidasan University,
Tiruchirappalli- 620 024, India.

[6] Department of Food Science and Nutrition,
College of Food Science and Agriculture,
King Saud University, Riyadh,
Kingdom of Saudi Arabia.

[7] Lee Kong Chian School of Medicine,
Nanyang Technological University, Singapore – 637553.

***Correspondence authors:**

[1] Dr. G. Archunan, Professor
Department of Animal Science,
Bharathidasan University, Tiruchirappalli – 620 024
Tamil Nadu, India, Phone: +91-431-2407040(Off),
Mobile: +91-9443922228, Fax: +91-431-2407045/2412750
Email: archunan@bdu.ac.in

[2] Dr. P. Padmanabhan, Senior Research Scientist
Lee Kong Chain School of Medicine
Nanyang Technological University
Singapore-637553, Phone: +65 93873979
Email: ppadmanabhan1@gmail.com or ppadmanabhan@ntu.edu.sg

Supplementary Figures and Tables:

Supplementary information Figure S1. Phosphorylation site and logo generation with conservation analysis. (A) Kinase specific eukaryotic protein phosphorylation sites (B) The functional conserved residues were identified from EULP using Conseq tool (C) The conserved sites were indicated in the 3D model.

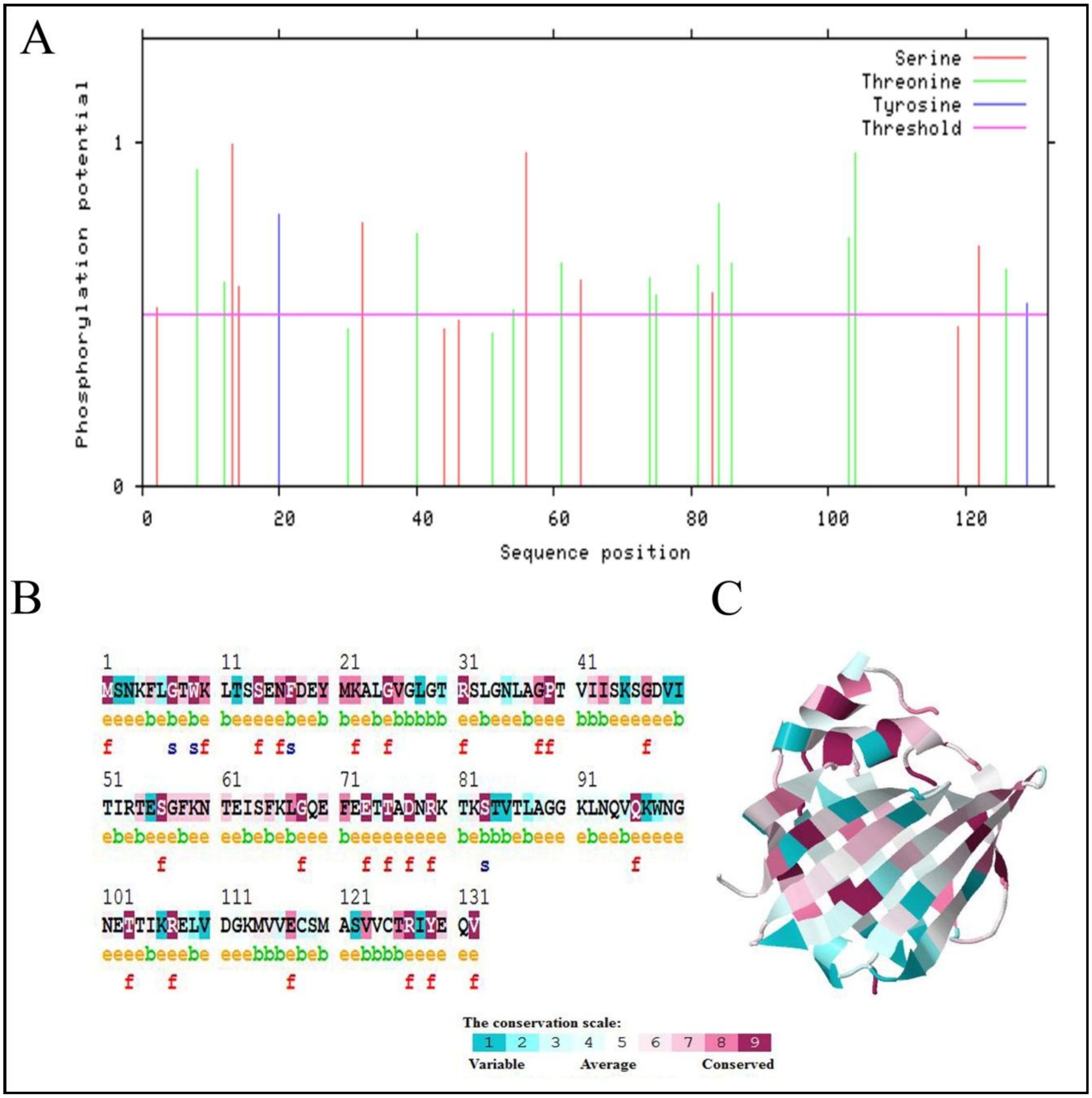

Supplementary information Figure S2. Topology prediction and transmembrane segment prediction of EULP. (A) H-bond interaction predicted in topology of EULP. (B)TMHMM (C) TMPRED (D) TOPPRED (E) Phobius.

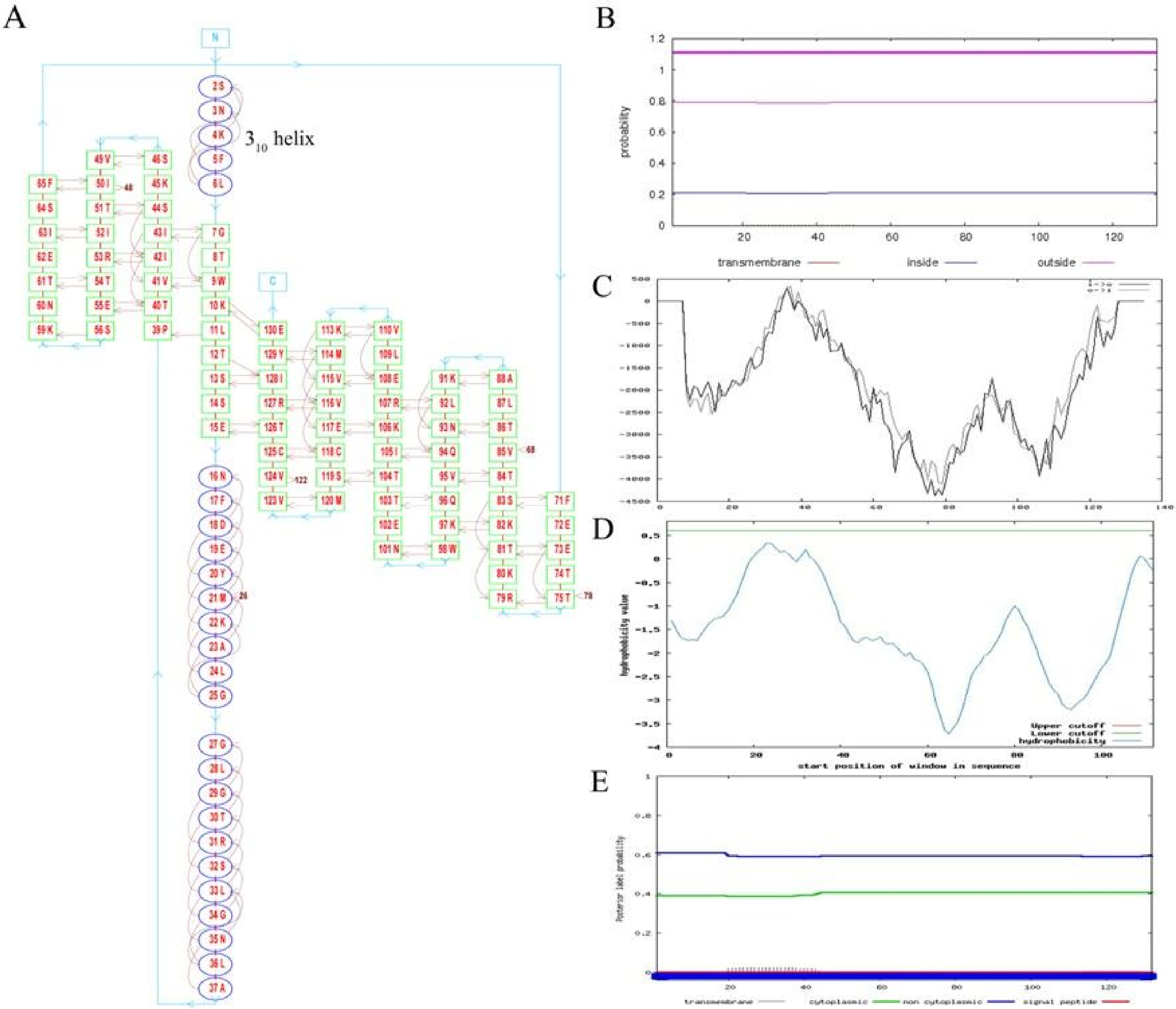

Supplementary information Figure S3. (A) The SDS- PAGE of female rat urinary protein profile. (M)- Molecular marker set proteins. (31-70) fractions collected from size exclusion chromatography. Each lane contains 30 µg of protein. The fractions from 59-63 were used for the florescent study. (B) Quenching experiments. The graph showed that the 2-napthol quenched by KI and the fluorescent ratio F_0/F. (C) 3D structure comparison of all compounds using tanimoto similarity analysis.

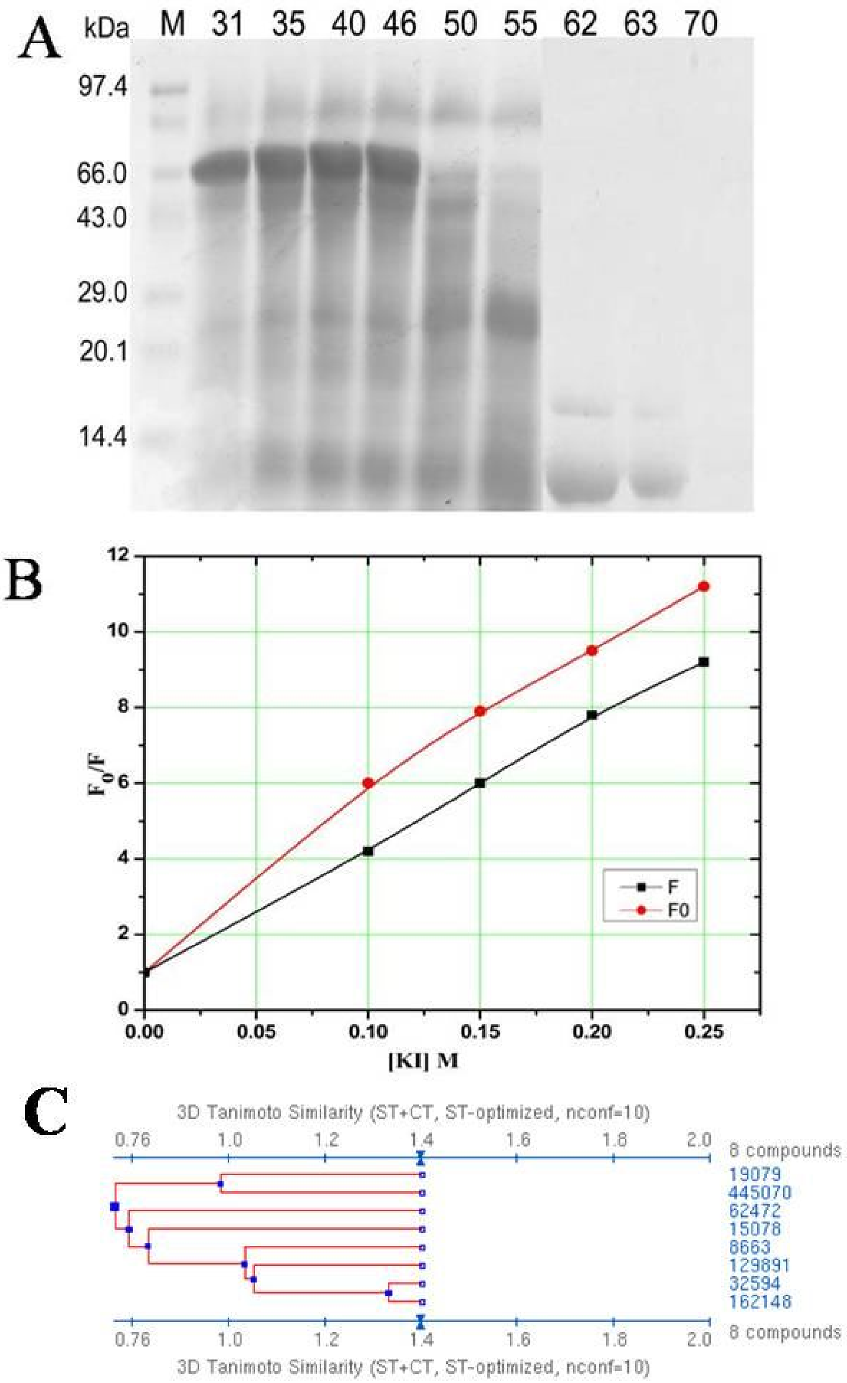

Supplementary Table S1. Log size, uniqueness and dispensability values of selected GO enriched proteins. The protein-protein interaction network was constructed based on molecular function of EULP.

S. No	Name	Log size	Uniqueness	Dispensability
**1**	chemokine (C-C motif) ligand 19 binding	1.556	0.785	0.707
**2**	ligase inhibitor activity	1.653	0.939	0.514
**3**	interleukin-6 binding	1.833	0.781	0.303
**4**	oleic acid binding	1.845	0.82	0.492
**5**	G-protein coupled serotonin receptor binding	1.863	0.798	0.46
**6**	lipopeptide binding	1.978	0.822	0.498
**7**	IgA binding	2.037	0.813	0.309
**8**	fatty acid derivative binding	2.267	0.932	0.025
**9**	apolipoprotein binding	2.477	0.807	0.323
**10**	pheromone binding	2.52	0.932	0.025
**11**	lipoprotein particle receptor activity	2.72	0.876	0.5
**12**	lipopolysaccharide receptor activity	2.748	0.873	0.503
**13**	chemokine binding	2.78	0.767	0.787
**14**	chemoattractant activity	2.804	0.96	0
**15**	lipase inhibitor activity	2.843	0.939	0
**16**	retinoid binding	2.856	0.811	0.544
**17**	fatty acid binding	2.858	0.807	0.124
**18**	lipoprotein particle receptor binding	2.862	0.782	0.509
**19**	protein-lipid complex binding	2.87	0.931	0.026
**20**	lipoprotein particle binding	2.87	0.931	0.026
**21**	steroid hormone receptor binding	3.175	0.777	0.027
**22**	glycolipid binding	3.232	0.801	0.566
**23**	glycoprotein binding	3.46	0.787	0.418
**24**	G-protein coupled chemoattractant receptor activity	3.585	0.857	0.574
**25**	phosphatidylinositol phosphate binding	3.626	0.796	0.653
**26**	steroid binding	3.7	0.799	0.622
**27**	lipid-transporting ATPase activity	3.798	0.892	0.304
**28**	fatty-acyl-CoA binding	3.866	0.954	0
**29**	G-protein coupled receptor binding	3.93	0.763	0.575
**30**	phospholipid transporter activity	3.949	0.907	0.799
**31**	fatty acid ligase activity	4.064	0.938	0
**32**	ligand-gated channel activity	4.296	0.93	0.252
**33**	protein kinase binding	4.401	0.773	0.403
**34**	phospholipase activity	4.607	0.935	0.416
**35**	lipid transporter activity	4.633	0.925	0
**36**	lipase activity	4.762	0.935	0.018

**37**	fatty acid synthase activity	5.024	0.956	0.016
**38**	lipid binding	5.049	0.921	0.054
**39**	drug binding	5.089	0.92	0.054
**40**	G-protein coupled receptor activity	5.093	0.838	0.77
**41**	transmembrane signaling receptor activity	5.244	0.843	0.797
**42**	receptor binding	5.293	0.753	0.554
**43**	ligase activity, forming carbon-oxygen bonds	5.73	0.938	0.489
**44**	signaling receptor activity	5.847	0.839	0
**45**	receptor activity	5.945	0.96	0
**46**	protein binding	6.07	0.914	0.042
**47**	small molecule binding	7.012	0.91	0.1
**48**	ion binding	7.197	0.912	0.199
**49**	Binding	7.42	0.982	0
**50**	Molecular_function	7.675	1	0

Supplementary Table S2. Conservation analysis of EULP sequence. The highly conserved and conserved residues of the protein identified using consurf tool.

Highly conserved (Grade 9)		Conserved (Grades 7-8)	Buried (B) /Exposed (E)
**Functional residue (E)**	**Structural residue (B)**		
**MET1**	GLY7	PHE5	B
**LYS10**	TRP9	ASP18	E
**SER14**	PHE17	TYR20	B
**ASN16**	SER83	MET21	B
**LYS22**		VAL26	B
**GLY25**		ILE42	B
**ARG31**		ILE43	B
**GLY38**		GLY57	E
**PRO39**		PHE58	B
**GLY47**		LYS59	E
**SER56**		ASN60	E
**GLY68**		THR61	E
**GLU73**		PHE65	B
**Thr75**		LYS67	B
**ASP77**		PHE71	B
**ARG79**		VAL123	B
**GLN96**		THR126	B
**THR103**			
**ARG107**			
**GLU117**			
**ARG127**			
**TYR129**			
**VAL132**			

Supplementary Table S3. The EULP active site residues were confirmed by CASTp analysis. The table shows the area and volumes of the 21 binding pockets were identified by molecular surface (MS) analysis in CASTp. The position of active sites residues are indicated.

Pocket	Area_MS	Vol_MS	Amino acid residues with position
21	1020.4	1323.15	PHE17,TYR20,MET21,LEU24,VAL26,THR30, ARG31,GLY34,ASN35,ALA37,PRO39,VAL41, **ILE52**,THR54,SER56,PHE58,LYS59,THR61, GLU62,ILE63,**PHE65**,**GLU73**,THR74,THR75, ALA76,ASP77,ARG79,**VAL85,LEU92**,**GLN94**, GLN96,**ILE105**,**ARG107**,VAL116,CYS118, ARG127,TYR129
20	53.76	57.57	GLU70,LYS82,SER83,THR84,VAL95,LYS97
19	89.33	63.18	LYS4,PHE5,TRP9,GLU108,LEU109,MET114
18	90.26	86.52	**MET1,SER2,LYS45**,**GLY47,ASP48,ILE50**,**LEU67**
17	64.27	61.21	LYS91,LYS106,ARG107,GLU108,VAL115,GLU117
16	83.33	61.33	**SER2,PHE5,ILE50,LEU67,LEU87,LEU92**
15	34.7	28.62	SER44,**LYS45**,SER46,**VAL49**,THR51
14	37.08	25.88	THR40,ILE42,ARG53,GLU55
13	41.19	23.16	THR104,LYS106,GLU117,SER119
12	31.78	16.59	GLY7,THR8,TRP9,GLN131
11	18.03	11.56	GLU72,LYS80,THR81,LYS82
10	36.64	22.52	MET1,LYS4,PHE5,LEU87,GLY90
9	24.91	17.17	THR84,THR86,ASN93,VAL95
8	18.69	10.46	ILE42,SER44,THR51
7	44.23	21	SER2,LYS45,ILE50,LEU67
6	17.29	10.18	GLU72,GLU73,THR74,LYS80
5	31.22	15.85	VAL116,CYS118,CYS125,ARG127
4	27.62	13.63	GLN94,GLN96,THR103,THR104,ILE105
3	33.69	14.73	PHE5,ILE43,ILE50,LEU92
2	22.84	11.87	LYS10,GLN131,VAL132
1	10.75	5.53	ASP18,LEU28,ARG31,SER32

Supplementary Table S4. Ligands are categorized by compound category and additional data for state, solubility and odour properties.

S. No	Pubchem ID	State	Solubility	Odour	Compound category			
					Kingdom	Super class	Class	Descriptors
**1**	CID_445070	Solid	Soluble in alcohol, Insoluble in water	Flowery and Citrus-Lime Odour	Organic compounds	Lipids	Fatty Acid Derivatives	Acyclic Alkenes
**2**	CID_32594	Liquid	Soluble in Organic solvents, Insoluble in water	Green bell pepper and vegetative aromas	Organic compounds	Aromatic Heteromonocyclic Compounds	Ethers	Pyrazines
**3**	CID_19079	Liquid	Soluble in Ethanol, Insoluble in water	Flavoring agents	Organic compounds	Aliphatic Acyclic Compounds	Thioethers	Primary Alcohols
**4**	CID_162148	Liquid	Soluble in water partially	Urinary odour	Organic compounds	Aromatic Heteromonocyclic Compounds	Thiazolines	Ketones
**5**	CID_129891	Solid	Soluble in Organic solvents, Insoluble in water	Green odour	Organic compounds	Organooxygen Compounds	Alcohols and Polyols	Ketones; Polyamines
**6**	CID_15078	Solid	Water soluble	Urinary odour	Organic compounds	Organoheterocyclic compounds	Triazines	Primary aromatic amine
**7**	CID_62472	Liquid	Soluble in water and readily soluble in ether	Green odour	Organic compounds	Aliphatic Acyclic Compounds	-	Peroxy Acids

Supplementary Table S5. Physico-chemical properties of selected ligands. General properties of all the ligands were data mined including canonical smiles, LogKow value and rule of violation from various chemical databases.

Pubchem ID	Canonical Smiles	H-bond donor	H-bond acceptor	Topological polar surface area (A^2)	Density (g/cm^3)	Boiling point (°C at 760 mmHg)	Melting point (°C)	Octanol/ Water Partition Coefficient (Log Kow est.)	Rule of violation
**CID_445070**	CC(=CCCC(=CCCC(=CCO)C)C)C	1	1	20.23	0.9±0.1	283.37	<25	4.39	0
**CID_32594**	CC(C)CC1=NC=CN=C1OC	0	3	35	1.0±0.1	210.8±35.0	45.12	2.86	0
**CID_19079**	CCCCCCCCSCCO	1	1	45.5	0.9±0.1	290.4±13.0	39.13	3.64	0
**CID_162148**	CCC(C)C1=NCCS1	0	2	37.7	1.06	193.033	29.18	4.03	0
**CID_129891**	CCC(=O)CCC(C)(C)O	1	2	37.3	0.929	230.92	12.67	0.64	0
**CID_15078**	C1(=NC(=NN1)N)N	3	4	93.6	1.5±0.1	473.7±28.0	325	-1.03	0
**CID_62472**	CCOO	1	2	29.5	0.949	95	-61.04	0.69	0
